# The Impact of Sickle Cell Disease on Academic Performance among Affected Students

**DOI:** 10.3390/children9010015

**Published:** 2021-12-27

**Authors:** Abdulaziz Alhazmi, Khalid Hakami, Faisal Abusageah, Essa Jaawna, Meshal Khawaji, Essam Alhazmi, Basem Zogel, Salman Qahl, Ghadeer Qumayri

**Affiliations:** 1 Department of Microbiology and Parasitology, College of Medicine, Jazan University, Jazan 45142, Saudi Arabia; 2 Emerging and Epidemic Infectious Diseases Research Unit, Medical Research Center, Jazan University, Jazan 45142, Saudi Arabia; 3 College of Medicine, Jazan University, Jazan 45142, Saudi Arabia; hakamikhaled5@gmail.com (K.H.); Abusageah.F.H@gmail.com (F.A.); EissaMJ@outlook.com (E.J.); Khawaji.M.J@gmail.com (M.K.); e.alhazmi1420@gmail.com (E.A.); Basem14201@hotmail.com (B.Z.); Salmanvip2020@hotmail.com (S.Q.); ghadeer.qumayri@gmail.com (G.Q.)

**Keywords:** sickle cell disease, hemoglobinopathies, Saudi Arabia, Jazan

## Abstract

Background: Sickle cell disease (SCD) is a genetic disease that is highly prevalent in Jazan Province, Saudi Arabia, and is mostly characterized by many complications such as vaso-occlusive crises (VOC), acute chest syndrome (ACS) and well-documented neurological complications. These complications may affect patients’ academic performance. Methods: An observational, cross-sectional, retrospective study was conducted in Jazan Province. General and demographic data were collected and questions about academic performance of students with SCD were answered. Both *t*-tests and chi-square tests, along with multiple logistic regression, were used for analysis. Results: 982 participants were selected for this study with a mean age of 23 years (SD: 7). Most of the participants were female (64%). The number of participants with SCD was 339 (36%), of whom 42% were male. Students with SCD recorded lower grade point averages (GPA) and more absences compared to healthy participants. Further, about 60% of students with SCD thought they performed better than 40% of the participants without SCD during the COVID-19 pandemic when most of the educational activities were online. Conclusion: As has been previously reported, this study suggested that the academic performance of students with SCD is negatively affected compared to healthy individuals, and this is mostly due to complications associated with the disease. Further, students with SCD acknowledged better performance with online education, an option that should be considered to improve their academic performance. National studies on a larger population are required by health and education officials, and supportive online educational programs are warranted to enhance the academic performance of this population.

## 1. Introduction

Sickle cell disease (SCD) is a genetically inherited hematological disorder that affects red blood cells (RBCs) and results in abnormal hemoglobin (Hb) [1,2,3]. Sickle cell anemia (SCA) is the most common and severe hereditary form of SCD [1,3]. Affected individuals are more likely to experience painful episodes known as vaso-occlusive crisis (VOC), which are considered a hallmark of the disease and which occur due to occlusion of small blood vessels by abnormally shaped RBCs [1]. Some complications that can occur are chronic hemolytic anemia, acute chest syndrome (ACS), cerebrovascular accident (CVA), repeated infections, hypoxia, and growth impairment [4]. According to the Center for Disease Control and Prevention (CDC), SCD affects millions of people worldwide and is more common in sub-Saharan Africa, Saudi Arabia, India, south and central America, and the Mediterranean countries [5]. The estimated number of SCD patients living in the United States of America (USA) in 2020 was approximately 100,000 [6].

The Middle Eastern and North African regions have a higher prevalence of SCD than the rest of the world [3] due to multiple factors, such as consanguineous marriages among first-degree relatives, lack of effective screening programs, and malaria [5,6]. In Africa, it is estimated that about 12 to 15 million have SCD [7]. In addition, approximately 75% to 85% of children born with SCD are born in different regions of Africa with a mortality rate that ranges from 50% to 80% for affected children under the age of 5 years [7,8]. In the Middle East, the highest prevalence of SCD and sickle cell trait (SCT) has been reported in the eastern region of Saudi Arabia (2.6% for SCD, and 2% to 27% for SCT, respectively) followed by Jazan [9,10]. Jazan Province, in south-western Saudi Arabia, is considered one of the most affected regions by this disease. It was found in a study carried out in 2020 that the complications associated with SCD, such as VOC and ACS, were the most common cause of hospital admission in Jazan [10]. CVA, VOC, growth impairment, in addition to central nervous system (CNS) complications, such as stroke, silent cerebral infarction, and cognitive impairment, could occur in students with SCD and negatively impact their academic performance, leading to academic achievement challenges, fewer career options, and in some people could have psychological and social impacts [11]. Thus, in this study, we aim to evaluate the academic performance of students with SCD compared to healthy participants, and to assess factors associated with poor academic performance.

## 2. Methods

We conducted this study using a cross-sectional observational design in Jazan Province located in the southwest corner of Saudi Arabia. The province harbors almost 1.7 million inhabitants [12]. We included all students of male and female sex in Jazan Province who had already been diagnosed with SCD and enrolled in middle school, high school, or university. A question was directed to the participants about whether they had been diagnosed with SCD. Individuals with no history of SCD were allowed to continue the survey for comparison purposes. Our exclusion criteria included individuals’ having other comorbidities, being primary school students, and being outside of Jazan Province. Data were primarily collected between August and September 2021 through an online anonymous self-administrated questionnaire designed via Google Forms to be filled by the participants. The distribution of the survey was based on the networks of the data collectors and investigators. The questionnaire was designed after an extensive review of the literature for related studies and after consulting experts in the field [4,13,14,15]. The questionnaire started with demographic questions about matters such as age, gender, current education level, parents’ monthly income, socio-economic status, level of parents’ education, and whether they had been previously diagnosed with SCD. Following this, data regarding academic performance, absence and attendance in the last three years were collected. Finally, participants were asked if their academic performance had been affected during COVID-19, during which period the educational activities were conducted online. Before the distribution of the survey, a pilot sample (*n* = 20) was used to assess the clarity and wording of questionnaire items. Data from this pilot sample were excluded from the analysis. For the sample size, we assumed that there were about 4500 patients with SCD in Jazan Province [16]. The sample size for this study was calculated using the Raosoft sample size calculator (Raosoft Inc., Seattle, WA, USA, raosoft.com) and 351 patients with SCD were needed to reach a 95% confidence interval and 5% margin of errors. The sampling design used was a simple random sampling technique, where we selected students with SCD from the whole group of participants. Descriptive statistics were reported for the collected data. Both the chi-square and the t-test were performed, and a multivariate analysis was performed using SPSS v.23 (IBM Corp., Armonk, NY, USA), with the alpha criterion for *p*-value set at 0.05. Ethical approval for conducting this study was obtained from the ethical approval committee at Jazan University (reference number: REC42/1/087; date: 22 March 2021). Written consent was obtained from all participants or their tutors prior to participation in the study.

## 3. Results

The survey was completed by a total of 928 participants who met our inclusion criteria. Most of the participants were female (64%), with a mean age of 23 (SD: 7). More than two-thirds of the participants were currently enrolled at the university, with 21% and 10% in high school and middle school, respectively. About 36% of the participants had been diagnosed with SCD (*n* = 339). More than half of the participants (either with or without SCD) recorded excellent performance based on their GPA for the previous three years, between 2018 and 2021. Further, 48% of the participants reported missing between 7 to 14 days in the 2019–2020 academic years, while 33% and 19% reported missing fewer than 7 days and more than 14 days, respectively. These data are summarized in Table 1. We analyzed the data from the participants who had been diagnosed with SCD (36%, *n* = 339) (Table 2). Most of the participants with SCD were female (58%), with a mean age of 21 (SD: 6). Half of the participants with SCD were currently enrolled at the university. The academic performance of participants with SCD between 2018 and 2021 seemed variable, with more than one-third of them having either excellent or very good performance, based on their GPA. About 40% of the participants with SCD reported missing more than 14 days in the last year and half of them acknowledged better academic performance during the COVID-19 pandemic, as most academic activities were conducted online. In Table 2, using univariate analysis, we compared participants who had been diagnosed with SCD with otherwise healthy individuals. We found the participants with SCD to be significantly younger compared to those without SCD (21 vs. 24 years, *p* = 0.0001). Further, we had more male participants in the SCD group compared to the healthy group (42% vs. 32%). A significant difference was found between participants with SCD and the healthy group regarding their city of residence (Table 2). Regarding parents’ education level and monthly income, no significant difference was recorded. Current education level was significantly different between the two groups (*p* = 0.0001 *). Moreover, the healthy group significantly recorded better academic performance in 2018, 2019 and 2021 (*p* = 0.0001, *p* = 0.003 and *p* = 0.025, respectively), and they significantly reported missing fewer days, compared to participants with SCD (*p* = 0.0001). During the COVID-19 pandemic, the SCD group answered that they had better academic performance (59% vs. 42%) compared to the healthy group (*p* = 0.0001). A multivariate analysis was performed for variables associated with SCD (Table 2). In Table 3, cumulative GPAs between 2018 and 2021 were calculated and analyzed for the participants with SCD. Female and younger participants had better academic performance. Further, better academic performance was associated with higher educational level and higher monthly income for parents. Participants with better academic performance reported missing fewer days and acknowledged better performance during the COVID-19 pandemic.

## 4. Discussion

SCD is a genetic condition characterized by sickle-shaped RBCs due to abnormal Hb formation. It causes a shorter lifespan by decreasing oxygen sufficiency of RBCs. Consequently, abnormal Hb and sickle-shaped RBCs harm different organs and cause various complications [1]. This chronic illness necessitates frequent use of health services at many levels: screening, immunizations, medications, blood transfusions, and hospitalizations for various reasons, such as VOC, ACS, and repeated infections. All these measures are in place to reduce associated morbidity and mortality [17]. Jazan Province has one of the highest prevalences of SCD in the country. Alsaeed et al. reported that the prevalence of SCD in Jazan was about 7% in 2017, with VOC and ACS as the major causes for hospital admission for 56% and 12%, respectively, of this total [18]. Despite this prevalence rate, no study in the region had assessed the academic performance of SCD patients. Thus, in this study, we aimed to fill this gap in knowledge, as we found that the academic performance of participants with SCD was significantly lower than that of healthy participants during the reported years from 2018 to 2021 (Table 2). These results are in line with those from another study conducted in Yemen in 2016 [13], which found a correlation between SCD severity and the academic performance of the affected students. Another study in Iraq, conducted in 2019, reported the same findings: students with SCD recorded lower performance compared to their classmates [14]. Lower school performance is usually associated with absenteeism among students with SCD. In our study, we found that 43% of the participants with SCD were absent for more than 14 days per annum, compared to 11% of the healthy participants in the 2019 academic year, i.e., before the COVID-19 pandemic (Table 2). In our multivariate analysis, healthy students significantly reported missing fewer days compared to the students with SCD (Table 2), and school absenteeism was significantly associated with poorer performance among students with SCD (Table 3). This result is consistent with Al-Saqladi’s data reported from Yemen; more than 60% of the study participants were absent for more than 20 days between 2013 and 2014 [13]. In another study conducted between 2015 and 2016, Abid et al. found that students with SCD missed significantly more school days, with an average of 12 days, compared to the healthy participants (average: 3 days, *p* < 0.001) [14]. Similarly, in 2016, Olatunya et al. conducted a study in Nigeria and concluded that a lower Hb rate is significantly associated with a higher absence rate [15]. On the other hand, Olusoga et al. found no association between school absenteeism and academic performance of students with SCD. Notably, this was study was conducted in 2005 in Nigeria, and the sample size did not exceed 52 students with SCD [4]. Thus, we believe that the larger sample size used in our study elaborated the association between school absenteeism and academic performance for students with SCD [4]. Epping et al. reviewed the medical records for 197 students with SCD and compared their academic performance with those of their healthy classmates. They concluded that students with SCD are not at high risk for academic difficulties, since children with SCD benefited from the individualized educational programs (IEP) they frequently received [2]. Thus, these kinds of programs are urgently needed in our region and the lack of these programs could explain the significant correlation between school absenteeism and lower performance for students with SCD in the current study and other studies in the region [13,14]. Taken together, these findings elucidate the challenges that students with SCD encounter and necessitate that education officials in the concerned countries take these challenges into consideration, since they add another burden that could negatively affect the quality of life of students with SCD [2,19].

In our study, it seems that students with SCD recorded better performance during the 2021 academic year compared to their performance in 2019 (the percentage of SCD students with excellent GPAs in 2019 and 2021 was 45% and 54% respectively, *p* = 0.01) (Table 2). During this year, most of the educational activities in Saudi Arabia were held online due to restrictions during the COVID-19 pandemic [20]. Furthermore, almost 60% of the students with SCD acknowledged that they did better during the COVID-19 pandemic, compared to 41% of the healthy students (Table 2), and this was associated with a higher GPA among students with SCD (Table 3). These findings suggest that online IEP could lead to better performance and limit factors that might affect the academic performance of students with SCD, such as place of residence or direct and indirect disease complications [21].

Place of residence appears to have a significant effect when comparing participants with and without SCD (Table 2), and this may be due to consanguineous marriages that traditionally followed in some conservative tribes in Jazan Province and exceeded 55% in some regions of Saudi Arabia [22,23]. Memish et al. have evaluated the outcome over the six years (2004 to 2009) of the premarital screening programs that have been applied. They found that the prevalence of beta-thalassemia was markedly decreased, while the prevalence of sickle cell disease was constant. They concluded that a noticeable reduction was recorded in the number of at-risk marriages [24]. Despite this conclusion, the incidence of SCD in Jazan is still elevated, and follow-up programs and studies are highly warranted to ensure better compliance with the premarital screening program and counseling recommendations [2]. Also, other socioeconomic factors are significantly associated with poorer academic performance among students with SCD, such as parent education level and monthly income (Table 3). This result is consistent with various reports that correlated parents’ education, monthly income and chronic diseases that may affect children at a younger age and negatively affect their academic performance [25,26].

This study is the first to assess the academic performance of students with SCD in Jazan Province and one of the few such studies in Saudi Arabia, a country with a large burden of hemoglobinopathies in general and SCD specifically. We believe that this study may help to pave the way for IEP that can be conducted online for students with SCD, which our findings suggest resulted in a better performance for students with SCD during the COVID-19 pandemic, a period in which most of the educational courses were online. However, this study possesses many limitations. It was based on an online survey that relies on the networks of the investigator and data collectors, and may have led to a non-response bias due to the barrier of Internet accessibility in some areas and among a certain type of population. In addition, the questionnaire failed to include a question related to SCD treatment adherence or factors of severity and their direct impact on academic performance. Further, with such a methodology, we would not be able to confirm the disease status of our participants or their GPA. However, we believe that we include real regional data of students with SCD in the Jazan Province and patients’ viewpoints of their disease condition and the manner in which it could affect their academic performance.

## 5. Conclusions

In this study, we found that the academic performance of students with SCD was negatively affected, and that they reported missing more days, compared to healthy students. These findings may be related to direct and indirect disease-related complications. Factors associated with better academic performance among participants with SCD are significantly related to female sex, younger age, having parents of higher education level and monthly income, and reporting fewer days missed and an acknowledged better performance during the COVID-19 pandemic. National studies on a larger population using official records are warranted and utilizing online IEP for students with SCD could result in a better performance for this group.

## Figures and Tables

**Table 1:** General characteristics of the participants in our study.

Variable	Participants, *n* = 928
Age, years (mean; SD)	23; 7
Male, *n* (%)	333 (36%)
Residence, *n* (%)	
Jazan	112 (12%)
Abu Arish	122 (13%)
Sabya	146 (16%)
Samtah	217 (23%)
Ahad Almasarhah	138 (15%)
Damad	64 (7%)
Farasan	4 (0.25%)
Faifa	9 (1%)
Alardah	35 (4%)
Ahurrath	4 (0.25%)
Addair	8 (1%)
Ad drab	14 (1.5%)
Baish	39 (4%)
Alaidabi	16 (2%)
Father’s highest education, *n* (%)	
Uneducated	107 (12%)
High school	357 (38%)
University level	415 (45%)
Postgraduate level	49 (5%)
Mother’s highest education, *n* (%)	
Uneducated	276 (30%)
High school	347 (37%)
University level	279 (30%)
Postgraduate level	26 (3%)
Monthly income (SAR), *n* (%)	
Less than 3000	208 (22%)
3000 to 5000	224 (24%)
5000 to 10,000	172 (19%)
10,000 to 20,000	176 (19%)
20,000 to 20,000	90 (10%)
More than 30,000	58 (6%)
Current education level, *n* (%)	
Middle school	95 (10%)
High school	195 (21%)
University	638 (69%)
Have been diagnosed with sickle cell anemia, *n* (%)	
No	405 (64%)
Yes	339 (36%)
GPA for 2018–2019 academic year, *n* (%)	
Excellent	488 (53%)
Very good	294 (32%)
Good	104 (11%)
Acceptable	26 (3%)
Weak	4 (1%)
Fail	12 (1%)
GPA for 2019–2020 academic year, *n* (%)	
Excellent	519 (56%)
Very good	273 (29%)
Good	103 (11%)
Acceptable	15 (2%)
Weak	5 (1%)
Fail	13 (1%)
GPA for 2020–2021 academic year, *n* (%)	
Excellent	540 (58%)
Very good	251 (27%)
Good	102 (11%)
Acceptable	14 (1%)
Weak	6 (1%)
Fail	15 (2%)
Reported missing school days between 2019 and 2020?	
Less than 7 days	303 (33%)
Between 7 and 14 days	448 (48%)
More than 14 days	177 (19%)
Is your academic performance affected during COVID-19?	
Not affected	303 (33%)
Yes, better performance	448 (48%)
Yes, worse performance	177 (19%)

SD: Standard deviation. SAR: Saudi Riyals.

**Table 2:** Comparison between healthy participants and participants with SCD.

	Univariate Analysis ^&^			Multivariate Analysis ^&&^	
Variable	Participants without SCD = 589 (64%)	Participants with SCD = 339 (36%)	*p*-Value ^#^	aOR	95% CI
Age, years (Mean; SD)	24; 7	21; 6	0.0001 *	0.961 **	0.936–0.988
Male, *n* (%)	191(32%)	142 (42%)	0.002 *	-	-
Residence, *n* (%)					
Jazan	99 (17%)	13 (4%)	0.0001 *	0.134 **	0.035–0.512
Abu Arish	67 (11%)	55 (16%)		-	-
Sabya	96 (16%)	50 (15%)		-	-
Samtah	153 (26%)	64 (19%)		-	-
Ahad Almasarhah	67 (11%)	71(21%)		-	-
Damad	34 (6%)	30 (9%)		-	-
Farasan	3 (0.5%)	1 (0%)		-	-
Faifa	4 (0.5%)	5 (1%)		-	-
Alardah	18 (3%)	17 (5%)		-	-
Ahurrath	1 (0.5%)	3 (1%)		-	-
Addair	4 (0.5%)	4 (1%)		-	-
Ad drab	13 (2%)	1 (0%)		0.580 **	0.005–0.645
Baish	23 (4%)	16 (5%)		-	-
Alaidabi	7 (2%)	9 (3%)		-	-
Father’s Highest education, *n* (%)					
Uneducated	68 (12%)	39 (12%)	0.779	-	-
High school	225 (38%)	132 (39%)		-	-
University level	268 (46%)	147 (43%)		-	-
Postgraduate level	28 (5%)	21 (6%)		-	-
Mother’s Highest education, *n* (%)					
Uneducated	166 (28%)	110 (32%)	0.398	-	-
High school	222 (38%)	125 (37%)		-	-
University level	186 (32%)	93 (28%)		-	-
Postgraduate level	15 (3%)	11 (3%)		-	-
Monthly income (SAR), *n* (%)					
Less than 3000	131 (22%)	77 (23%)	0.49	-	-
3000 to 5000	150 (25%)	74 (22%)		-	-
5000 to 10,000	100 (17%)	72 (21%)		-	-
10,000 to 20,000	114 (19%)	62 (18%)		-	-
20,000 to 20,000	60 (10%)	30 (9%)		-	-
More than 30,000	34 (6%)	24 (7%)		-	-
Current education level, *n* (%)					
Middle school	34 (6%)	61 (18%)	0.0001 *	3.251 **	1.851–5.708
High school	99 (17%)	96 (28%)		2.580 **	1.674–3.977
University	456 (77%)	182 (54%)		-	
GPA for 2018-2019 academic year, *n* (%)					
Excellent	337 (57%)	151 (45%)	0.0001 *	-	-
Very good	184 (31%)	110 (32%)		-	-
Good	57 (10%)	47 (14%)		-	-
Acceptable	6 (1%)	20 (6%)		-	-
Weak	1 (0%)	3 (1%)		-	-
Fail	4 (1%)	8 (2%)		-	-
GPA for 2019-2020 academic year, *n* (%)					
Excellent	350 (59%)	169 (50%)	0.003 *	-	-
Very good	172 (29%)	101 (30%)		-	-
Good	54 (9%)	49 (14%)		-	-
Acceptable	7 (1%)	8 (2%)		-	-
Weak	1 (0%)	4 (1%)		-	-
Fail	5 (1%)	8 (2%)		-	-
GPA for 2020-2021 academic year, *n* (%)					
Excellent	358 (61%)	182 (54%)	0.025 *	-	-
Very good	160 (27%)	91 (27%)		-	-
Good	55 (9%)	47 (14%)		-	-
Acceptable	7 (1%)	7 (2%)		-	-
Weak	4 (1%)	2 (1%)		-	-
Fail	5 (1%)	10 (3%)		-	-
Reported missing school days between 2019 and 2020?					
Less than 7 days	376 (64%)	75 (22%)	0.0001 *	0.089 **	0.056–0.140
Between 7 and 14 days	150 (25%)	119 (35%)		0.340 **	0.217–0.532
More than 14 days	63 (11%)	145 (43%)		-	-
Did your academic performance was affected during COVID-19?					
Not affected	218 (37%)	85 (25%)	0.0001 *	-	-
Yes, better performance	249 (42%)	199 (59%)		0.008 **	1.183–3.023
Yes, worse performance	122 (21%)	55 (16%)		-	-

SD: Standard deviation. SCD: Sickle Cell Disease. SAR: Saudi Riyals. ^#^ The alpha criterion for *p*-value was set to 0.05. * Significant in univariate analysis. ** Significant in Multivariate analysis. ^&^ Chi-squared test and *t*-test were used for univariate analysis. ^&&^ Multiple logistic regression was used for variable significantly associated with SCD in univariate analysis.

**Table 3:** Cumulative GPA between 2018 and 2021 for participants with SCD and associated factors.

Variable	Cumulative GPA between 2018 and 2021 for Participants with SCD						
	Excellent	Very Good	Good	Acceptable	Weak	Fail	*p*-Value ^#^
*n*	167	104	48	9	5	6	
%	49%	31%	14%	3%	1%	2%	
Age, years (Mean; SD)	20; 5	22; 7	22; 5	21; 5	30; 9	34; 5	0.0001 *
Sex, *n* (%)							
Male	54	49	25	4	5	5	0.001 *
	38%	35%	18%	3%	4%	4%	
Female	113	55	23	5	0	1	
	57%	28%	12%	3%	0%	1%	
Residence, *n* (%)							
Jazan	8	0	3	1	1	0	0.926
	62%	0%	23%	8%	8%	0%	
Abu Arish	26	17	10	0	1	1	
	47%	31%	18%	0%	2%	2%	
Sabya	26	17	5	0	0	2	
	52%	34%	10%	0%	0%	4%	
Samtah	27	19	11	4	2	1	
	42%	30%	17%	6%	3%	2%	
Ahad Almasarhah	38	22	9	1	0	1	
	54%	31%	13%	1%	0%	1%	
Damad	13	10	5	2	0	0	
	43%	33%	17%	7%	0%	0%	
Farasan	0	1	0	0	0	0	
	0%	100%	0%	0%	0%	0%	
Faifa	2	3	0	0	0	0	
	40%	60%	0%	0%	0%	0%	
Alardah	8	6	2	0	1	0	
	47%	35%	12%	0%	6%	0%	
Ahurrath	2	0	1	0	0	0	
	67%	0%	33%	0%	0%	0%	
Addair	3	0	1	0	0	0	
	75%	0%	25%	0%	0%	0%	
Ad drab	1	0	0	0	0	0	
	100%	0%	0%	0%	0%	0%	
Baish	7	7	1	0	0	0	
	47%	47%	6%	0%	0%	0%	
Alaidabi	6	2	0	1	0	1	
	60%	20%	0%	10%	0%	10%	
Father’s highest education, *n* (%)							
Uneducated	17	17	3	2	0	0	0.009 *
	44%	44%	8%	5%	0%	0%	
High school	56	41	23	4	2	6	
	42%	31%	17%	3%	2%	5%	
University level	76	55	21	3	2	0	
	48%	35%	13%	2%	1%	0%	
Postgraduate level	18	1	1	0	1	0	
	86%	5%	5%	0%	5%	0%	
Mother’s highest education, *n* (%)							
Uneducated	40	40	17	5	2	6	0.027 *
	36%	36%	15%	5%	2%	5%	
High school	62	37	21	3	2	0	
	50%	30%	17%	2%	2%	0%	
University level	58	24	9	1	1	0	
	62%	26%	10%	1%	1%	0%	
Postgraduate level	7	3	1	0	0	0	
	64%	27%	9%	0%	0%	0%	
Monthly income (SAR), *n* (%)							
Less than 3000	24	27	14	4	4	4	0.005 *
	31%	35%	18%	5%	5%	5%	
3000 to 5000	35	27	8	2	0	2	
	47%	36%	11%	3%	0%	3%	
5000 to 10,000	35	20	15	2	0	0	
	49%	28%	21%	3%	0%	0%	
10,000 to 20,000	40	14	7	1	0	0	
	65%	23%	11%	2%	0%	0%	
20,000 to 20,000	15	12	3	0	0	0	
	50%	40%	10%	0%	0%	0%	
More than 30,000	18	4	1	0	1	0	
	75%	17%	4%	0%	4%	0%	
Current education level, *n* (%)							
Middle school	30	21	6	2	1	1	0.114
	49%	34%	10%	3%	2%	2%	
High school	40	28	22	1	1	4	
	42%	29%	23%	1%	1%	4%	
University	97	55	20	6	3	1	
	53%	30%	11%	3%	2%	1%	
Reported missing school days between 2019 and 2020?							
Fewer than 7 days	49	15	11	0	0	0	0.001 *
	65%	20%	15%	0%	0%	0%	
Between 7 and 14 days	61	42	14	2	0	0	
	51%	35%	12%	2%	0%	0%	
More than 14 days	57	47	23	7	5	6	
	39%	32%	16%	5%	3%	4%	
Was your academic performance affected during COVID-19?							
Not affected	42	26	12	0	1	4	0.018 *
	49%	31%	14%	0%	1%	5%	
Yes, better performance	108	56	26	7	2	0	
	54%	28%	13%	4%	1%	0%	
Yes, worse performance	17	22	10	2	2	2	
	31%	40%	18%	4%	4%	4%	

SD: Standard deviation. SCD: Sickle cell disease. SAR: Saudi riyals. ^#^ The alpha criterion for the *p*-value was set to 0.05. * Significant in univariate analysis.

## Data Availability

The data presented in this study are available on request from the corresponding author.

## References

[1] CDC What Is Sickle Cell Disease?. https://www.cdc.gov/ncbddd/sicklecell/facts.html.

[2] Epping A.S., Myrvik M.P., Newby R.F., Panepinto J.A., Brandow A.M., Scott J.P. (2013). Academic Attainment Findings in Children With Sickle Cell Disease. J. Sch. Health.

[3] Pandarakutty S., Murali K., Arulappan J., Al Sabei S.D. (2020). Health-related quality of life of children and adolescents with sickle cell disease in the Middle East and North Africa region: A systematic review. Sultan Qaboos Univ. Med. J..

[4] Ogunfowora O.B., Olanrewaju D.M., Akenzua G.I. (2005). A comparative study of academic achievement of children with sickle cell anemia and their healthy siblings. J. Natl. Med. Assoc..

[5] CDC Data & Statistics on Sickle Cell Disease. https://www.cdc.gov/ncbddd/sicklecell/data.html.

[6] Piel F.B., Hay S., Gupta S., Weatherall D.J., Williams T.N. (2013). Global Burden of Sickle Cell Anaemia in Children under Five, 2010–2050: Modelling Based on Demographics, Excess Mortality, and Interventions. PLoS Med..

[7] Mulumba L.L., Wilson L. (2015). Sickle cell disease among children in Africa: An integrative literature review and global recom-mendations. Int. J. Afr. Nurs. Sci..

[8] Makani J., Cox S., Soka D., Komba A.N., Oruo J., Mwamtemi H., Magesa P., Rwezaula S., Meda E., Mgaya J. (2011). Mortality in Sickle Cell Anemia in Africa: A Prospective Cohort Study in Tanzania. PLoS ONE.

[9] Jastaniah W. (2011). Epidemiology of sickle cell disease in Saudi Arabia. Ann. Saudi Med..

[10] Hazzazi A.A., Ageeli M.H., Alfaqih A.M., Jaafari A.A., Malhan H.M., Bakkar M.M. (2020). Epidemiology and characteristics of sickle cell patients admitted to hospitals in Jazan region, Saudi Arabia. J. Appl. Hematol..

[11] Day S., Chismark E. (2006). The cognitive and academic impact of sickle cell disease. J. Sch. Nurs..

[12] Azalghamdi Population in Jazan Region by Gender, Age Group, and Nationality (Saudi/Non-Saudi). https://www.stats.gov.sa/en/6140.

[13] Al-Saqladi A.-W.M. (2016). The impact of sickle cell disease severity on school performance in affected Yemeni children. J. Appl. Hematol..

[14] Hassan M., Abid F.H., Ahmed B.A.A.H. (2019). School performance of children with sickle cell disease in Basra, Iraq. Iraqi J. Hematol..

[15] Olatunya O.S., Oke O.J., Kuti B.P., Ajayi I.A., Olajuyin O., Omotosho-Olagoke O., Taiwo A.B., Faboya O.A., Ajibola A. (2017). Factors Influencing the Academic Performance of Children with Sickle Cell Anaemia in Ekiti, South West Nigeria. J. Trop. Pediatr..

[16] Alotaibi M.M. (2017). Sickle cell disease in Saudi Arabia: A challenge or not. J. Epidemiol. Glob. Health.

[17] Kauf T.L., Coates T.D., Huazhi L., Mody-Patel N., Hartzema A.G. (2009). The cost of health care for children and adults with sickle cell disease. Am. J. Hematol..

[18] AlSaeed E.S., Farhat G.N., Assiri A.M., Memish Z., Ahmed E.M., Saeedi M.Y., Al-Dossary M.F., Bashawri H. (2017). Distribution of hemoglobinopathy disorders in Saudi Arabia based on data from the premarital screening and genetic counseling program, 2011–2015. J. Epidemiol. Glob. Health.

[19] Crosby L.E., Joffe N.E., Irwin M.K., Strong H., Peugh J., Shook L.M., Kalinyak K.A., Mitchell M.J. (2015). School Performance and Disease Interference in Adolescents with Sickle Cell Disease. Phys. Disabil. Educ. Relat. Serv..

[20] Algaissi A.A., Alharbi N.K., Hassanain M., Hashem A.M. (2020). Preparedness and response to COVID-19 in Saudi Arabia: Building on MERS experience. J. Infect. Public Health.

[21] Hassan K., AhmedQalawa S.A. (2021). Impact of Educational Program for Adolescents and Young Adults with Sickle Cell diseases on their knowledge, Perception, and Self-Care. Indian J. Public Health Res. Dev..

[22] El-Hazmi M.A., Al-Swailem A.R., Warsy A.S., Sulaimani R., Al-Meshari A.A. (1995). Consanguinity among the Saudi Arabian population. J. Med. Genet..

[23] Al-Gazali L., Hamamy H., Al-Arrayad S. (2006). Geneticdisorders in the Arab world. BMJ.

[24] Memish Z.A., Saeedi M.Y. (2011). Six-year outcome of the national premarital screening and genetic counseling program for sickle cell disease and β-thalassemia in Saudi Arabia. Ann. Saudi Med..

[25] Ong L.C., Chandran V., Lim Y.Y., Chen A.H., Poh B.K. (2010). Factors associated with poor academic achievement among urban primary school children in Malaysia. Singap. Med. J..

[26] Ozmert E.N., Yurdakok K., Soysal Ş., Kayikci M.K., Belgin E., Laleli Y., Saraçbaşi O. (2005). Relationship Between Physical, Environmental and Sociodemographic Factors and School Performance in Primary Schoolchildren. J. Trop. Pediatr..

